# OP57 Comparing Institute For Clinical And Economic Review Comparative Effectiveness Assessments And Federal Joint Committee Added Benefit Assessments

**DOI:** 10.1017/S0266462324001193

**Published:** 2025-01-07

**Authors:** Michael J. DiStefano, Steven D. Pearson, David M. Rind, Antal Zemplenyi

## Abstract

**Introduction:**

We compared the Institute for Clinical and Economic Review’s (ICER) ratings of comparative clinical effectiveness with the German Federal Joint Committee’s (G-BA) added benefit ratings, and explored what factors, including the evidence base, may explain disagreement between the two organizations.

**Methods:**

Drugs were included if they were assessed by ICER under its 2020–2023 Value Assessment Framework and had a corresponding assessment by G-BA as of March 2023 for the same indication, patient population, and comparator drug. To compare assessments, we modified ICER’s proposed crosswalk between G-BA and ICER benefit ratings to account for G-BA’s extent and certainty ratings. We also determined whether each assessment pair was based on similar or dissimilar evidence. Assessment pairs exhibiting disagreement based on the modified crosswalk despite a similar evidence base were qualitatively analyzed to identify reasons for disagreement.

**Results:**

We identified 15 assessment pairs and seven out of fifteen were based on similar evidence. G-BA and ICER assessments disagreed for each of these drugs. For 4/7 drugs, G-BA (but not ICER) determined the evidence was unsuitable for assessment: for 2/4 drugs, G-BA concluded the key trials did not appropriately assess the comparator therapy; for 1/4, G-BA did not accept results of a before-and-after study due to non-comparable study settings; for 1/4, G-BA determined follow-up in the key trial was too short. Among assessment pairs where both organizations assessed the evidence, reasons for disagreement included concerns about long-term safety, generalizability, and study design.

**Conclusions:**

This study underscores the role of value judgments within assessments of clinical effectiveness. These judgments are not always transparently presented in assessment summaries. The lack of clarity regarding these value-based decisions underscores the need for improvements in transparency and communication, which are essential for promoting a more robust health technology assessment process and supporting transferability of assessments across jurisdictions.

